# Health‐Promoting Schools in Israel, Applying the Ottawa Charter for Health Promotion

**DOI:** 10.1111/josh.70105

**Published:** 2026-01-04

**Authors:** Samira Obeid, Tikva Peretz, Shani Barzilai, Olga Winizki, Orna Baron‐Epel

**Affiliations:** ^1^ Department of Nursing The Max Stern Yezrael Valley College Yezrael Valley Israel; ^2^ Public Health Research Department North District, the Ministry of Health Nof Hagalil Israel; ^3^ Department of Nursing North District, Ministry of Health Nof Hagalil Israel; ^4^ Nutrition Department North District, Ministry of Health Nof Hagalil Israel; ^5^ Former District Medical Officer (Retired) North District, Ministry of Health Nof Hagalil Israel; ^6^ School of Public Health, University of Haifa Haifa Israel

**Keywords:** Arab, areas of action, health‐promoting schools (HPS), Jews, Ottawa charter, socioeconomic status

## Abstract

**Background:**

School‐based health promotion programs can improve children's health outcomes. This study examined levels of implementation of health promotion by Areas of Action in health‐promoting schools (HPS) and non‐HPS in Israel.

**Methods:**

A cross‐sectional survey was conducted among principals from 637 of 771 Israeli elementary and middle schools (52% Jewish, 48% Arab). The questionnaire assessed school activities according to Action Areas and domains of the Ottawa Charter.

**Results:**

HPS recognition rates were 64% in secular Jewish schools and 69% in Arab schools. Among non‐HPS schools, 54% (*n* = 164) reported health promotion activities. HPSs had higher activity levels than non‐HPSs in the domains of policy (M = 7.79 vs. 5.45), environment (7.78 vs. 6.85), health education (2.01 vs. 1.67), and staff training and empowerment (1.71 vs. 0.97), all *p* < 0.001. Community involvement was also higher (90.8% vs. 58.6%, *χ* ^2^ (1) = 62.92, *p* < 0.001. In Jewish schools, a higher socioeconomic status (SES) correlated with increased activity (*B* = 0.13, *β* = 0.107, *t* = 2.38, *p* < 0.05). Among Arab schools, SES was not significantly related to most domains, except for staff training and community involvement, where associations were negative.

**Implications for School Health Policy, Practice, and Equity:**

A comprehensive, equity‐oriented policy is needed to ensure equal opportunities for health promotion, particularly in underserved communities.

**Conclusions:**

Strengthening the implementation of the Ottawa Charter principles alongside formal recognition of HPSs is fundamental to advancing effective school health promotion.

## 1 Introduction

The Health‐Promoting Schools (HPS) framework, developed in the 1980s, has grown into a globally recognized network fostering student health‐enhancing behaviors [1]. According to the World Health Organization (WHO), a health‐promoting school continuously strengthens its capacity to be a healthy environment for living, learning, and working. It effectively encourages children to adopt healthy behaviors and reduce those that pose risks [2].

The Health Promoting Schools (HPS) framework, based on the WHO Ottawa Charter for Health Promotion, encompasses five core strategies: policy development for health promotion, environmental support systems, community program enhancement, individual skill building, and health service restructuring [2]. These approaches have evolved school health interventions from metric‐based assessments to comprehensive, collaborative models that engage entire school communities and external partners [3]. Emerging evidence demonstrates the application of Ottawa Charter strategies across diverse health promotion interventions. Yet research tends to adopt a selective approach, emphasizing specific strategies while neglecting comprehensive evaluation of the complete Charter framework. Recent research has examined health promotion interventions using Ottawa Charter strategies across various settings. Most studies focus on individual strategies rather than evaluating the complete five‐component framework [4, 5, 6]. Comprehensive evaluation of all five Ottawa Charter strategies remains limited in the literature. Tiraihati's qualitative investigation represents one of the few studies that examined the complete framework [7].

In Israel, the Ministries of Education and Health established a network of HPSs in the early 2000s, applying the WHO's international framework. Schools that join this network commit to promoting student health, enhancing the quality of life, and fostering wellbeing for all school community members. To join the network, schools must develop a multi‐year program integrated into the curriculum, focusing on healthy behaviors and lifestyles. This program must meet the health promotion standards of the Ministries of Education and Health, incorporating clear goals, content, activities, and evaluation processes [8].

National health‐promoting school policies vary significantly across countries. The 2013 Schools for Health in Europe (SHE) network survey revealed that formal policies existed in 62% of participating countries, frequently integrated within comprehensive educational reform initiatives [9]. In Israel, HPSs represent about 35% of educational institutions, spanning elementary to high schools and serving diverse ethnic and religious populations [10]. Guided by Ottawa Charter principles, these educational institutions embed health‐promoting policies within their broader institutional ethos and operational framework [11].

Schools are considered ideal settings for promoting health, as they directly influence students' quality of life and wellbeing [12]. However, the implementation of health programs faces significant barriers, including limited funding and resources and staffing shortages [13]. Other challenges include time constraints, heavy workloads, lack of training, and insufficient willingness to engage beyond the standard curriculum [14].

Comparative research in Israel has evaluated HPS outcomes versus conventional school approaches. Evidence suggests that Health Promoting Schools exhibit enhanced policy adoption for nutritional health and physical activity promotion compared to non‐designated institutions [15]. Contradictory evidence emerged from another investigation, which identified no substantial behavioral differences between HPS and non‐HPS student populations, implying limited efficacy of health promotion strategies in achieving targeted behavioral modifications [16]. Tesler et al. also found no direct linkage between policy execution and student behavioral changes, yet highlighted parental engagement in health initiatives as a crucial determinant of improved student results [17]. None of the studies reviewed reported on intervention programs based on a comprehensive application of all five Action Areas of the Ottawa Charter. Instead, most focused on one or several specific strategies, rather than adopting an integrated, holistic approach that addresses the full framework of health promotion. Hence, the current study is needed to address this gap by examining health promotion initiatives through a comprehensive lens that incorporates all five Action Areas of the Ottawa Charter.

In Israel in the Northern region, 421 out of 822 schools (51%) were recognized by the Ministry of Education as HPSs as of 2021. Despite this, studies show a significant disparity in health outcomes, such as higher rates of overweight and obesity among children in this region compared to other regions in Israel [18, 19]. All schools in this district receive health services from the Ministry of Health.

Therefore, this study examines: (1) the level of implementation of health promotion policies in the Northern District schools (2) how the Ottawa Charter Areas of Action (developing health‐promoting policies, creating supportive environments, strengthening community programs, enhancing individual competencies, and reorganizing health services) apply to the health promotion activities in schools (3) what the factors associated with health promotion activities in schools are?

## 2 Methods

### 2.1 Design and Setting

This is a cross‐sectional survey conducted from 1/09/2022 through 30/09/2022, targeting principals of all schools (elementary and middle) receiving health services from the Ministry of Health in the Northern District of Israel.

### 2.2 Participants

There are 822 schools in the Northern District run by the Ministry of Education, including 51 schools designated as special education institutions. The special education institutions were excluded from the study due to their unique characteristics. Of the remaining schools (771), 637 agreed to participate and were included in the study, representing 83% of all schools. Ninety‐two percent of these (586) were elementary schools, and the rest were middle schools. Fifty‐two percent (331) were Hebrew‐speaking schools, and 48% (303) were Arab‐speaking schools. The Hebrew‐speaking schools were divided into three groups: secular 72% (461), Jewish religious schools 22% (72), and ultraorthodox religious schools 14% (90). The Ministry of Education supervises all schools, and school nurses from the Ministry of Health provide public health services.

### 2.3 Procedure

School nurses interview the school principals or their representatives at the beginning of each school year. They conduct a basic assessment to describe the school's health profile. For this study, the school nurses were also asked to complete the research questionnaire while conducting these routine interviews with the school principals or their representatives.

### 2.4 Instrumentation

A quantitative questionnaire was developed based on the School Health Promotion Scale [20]. The questionnaire examined whether the school received recognition and belonged to the network of HPSs. It also assessed the engagement in health‐related activities among schools that had not been recognized as HPSs and included questions describing health promotion activities at the school, according to the five Areas of Action outlined in the Ottawa Charter. The validity and reliability of the questionnaire were examined using several methodologies. Content validity was established through expert review, wherein the questionnaire was submitted to field specialists including nursing directors with expertise in school health, the health coordinator from the Ministry of Education, the district nursing supervisor, a health promotion specialist, and the district medical officer. These domain experts evaluated the questionnaire's comprehensiveness and relevance to the research objectives, ensuring that it adequately captured the essential dimensions of the construct under investigation. Additionally, convergent validity was assessed by comparing the questionnaire items with those from other instruments that measured the application of Ottawa Charter Action Areas in various contexts. This comparative analysis involved examining established research frameworks that had previously operationalized Ottawa Charter principles in different health promotion settings, allowing for the identification of consistent measurement patterns across instruments [21]. Reliability assessment utilized Cronbach's alpha coefficient to evaluate variables representing school characteristics and activities across four Ottawa Charter domains: policy implementation, environmental factors, personal skill development, and community engagement, producing an overall reliability coefficient of 0.76. Pre‐testing involved interviews with 10 school principals to gather practical insights. Their comments and suggestions were systematically analyzed, and appropriate modifications were implemented in the final instrument. The school's SES variable was determined according to the SES index of the locality in which it is located. According to the Central Bureau of Statistics [22], classification, at the time of the survey, the socioeconomic ranking (SES) of these localities ranged from a minimum of two to a maximum of nine (out of 10), with an overall average of four and a standard deviation of 1.69.

The questionnaire is detailed in Table A1.

### 2.5 Data Analysis

Analysis was conducted using the IBM SPSS Statistics 27. We created variables reflecting the number of school features and activities that support each of the Ottawa Charter Areas of Action: policy, environment, developing personal skills, and community involvement.

Moreover, the scale for each Area of Action (policy, environment, developing personal skills, and community involvement) was also determined based on the number and type of questions. For example, the policy variable was assessed by 11 questions, with responses being either “yes” (indicating that health is included in the school's Charter) or “no.” Similarly, the environment variable was assessed using 12 questions in the same format. Both variables followed a normal distribution and were treated as continuous quantitative variables in further analysis.

The variable “Personal Skills Development” was examined through two components: health education by integrating health topics into educational programs, and training and empowerment programs for the teaching staff. In the analyses, each component was examined separately due to the fact that each one addressed a different target population. Neither of these variables followed a normal distribution; therefore, they were treated as ordinal or count variables.

The community involvement variable was examined using five questions that assessed whether or not the school engaged with the community, as well as by detailing with whom the school was involved. These included the involvement of parents, the participation of municipal departments related to health (e.g., environmental health), the involvement of health professionals living or working in the school's community, and the participation of additional bodies such as NGOs and volunteer groups.

For the exact wording of the questions, see Table A1. Table 1 describes the five variables.

**TABLE 1:** Descriptive statistics of health promotion areas of action variables.

	No. of items	Range	Med	M/%	SD	Type
Policy	11	0–11	7	6.61	2.18	Quantitative
Environment	12	0–11	7	7.30	2.10	Quantitative
Developing personal skills						
Health education	3	0–3	2	1.83	0.76	Count/Ordinal
Teaching staff training and empowerment	2	0–2	1	1.34	0.74	Count/Ordinal
Community involvement^a^	4	0–1	1			Count/Ordinal
Community involvement^b^	1	0–1	1	73.3%	—	Binary

^a^ Describes types of community involvement: parents, municipal departments, health professionals, NGOs and volunteer groups.

^b^ Describes the variable calculated according to the division involved/not involved.

Comparisons between HPSs and non‐HPSs, as well as Jewish and Arab schools, were performed using independent sample *t*‐tests and non‐parametric Mann–Whitney tests. One‐way ANOVA tests, Tukey's HSD post hoc tests, non‐parametric Kruskal–Wallis tests, and Bonferroni‐corrected post hoc, were used to examine whether public‐secular, public‐religious, and ultraorthodox Jewish schools differ in the amount of health promoting features and activities related to the five health‐promotion strategies.

## 3 Results

### 3.1 Health‐Promoting Schools and Non‐Health‐Promoting Schools

The survey results show that half of the schools in the Northern district have been recognized as HPSs in the past 3 years, totaling 50% (322).

Comparing the sectors of Jewish schools by religious affiliation, more than half the secular schools received recognition as HPSs 62% (282), compared to religious schools 28% (20), and ultraorthodox schools 14% (12); the difference was statistically significant, *p* < 0.01.

Moreover, in the Arab sector a higher percent of schools were recognized as HPSs 69% (209/303), compared to only 33% (109/331) in the Jewish sector. The difference was also statistically significant, *p* < 0.01.

Principals of schools that did not receive recognition as HPSs were asked if they engaged in health‐related activities. Data analysis showed that 54% of these schools (164) engage in health‐related activities. No differences were found between schools based on religious and ethnic affiliation.

HPSs had higher levels of activity in all Areas of Action of the Ottawa Charter, (see Table 2). Moreover, HPSs were more likely to involve the community in health‐promotive activities than non‐HPSs (*χ* ^2^_(1)_ = 62.92, *p* < 0.001). Community involvement activities occurred in 90.8% of HPSs versus 58.6% of non‐HPSs. Most HPSs involved parents (79%), health professionals (84%), and NGOs or volunteer organizations (70%) almost equally. No school reported involving local government departments. This variable is not reported in Table 2 because its measurement scale is different from the other variables in the table.

**TABLE 2:** Health promoting areas of action in HPS and non‐HPS: Means and standard deviation.

Areas of action	Non‐HPSs (*N* = 315)		HPSs (*N* = 322)			
	M	SD	M	SD	*t/z*	*d*
Policy	5.45	1.90	7.79	1.71	16.25*	1.29
Environment	6.85	2.07	7.78	1.96	5.85*	0.46
Developing personal skills						
Health education^a^	1.67	0.75	2.01	0.72	5.68*	0.46
Teaching staff training and empowerment^a^	0.97	0.73	1.71	0.52	12.96*	1.17

^a^ Non‐parametric comparison (Mann–Whitney test).

* *p* < 0.001.

### 3.2 Comparison Between Schools by Ethnic Affiliation and Religiosity

Table 3 presents the comparisons between Jewish and Arab schools. The study results indicated that Arab schools have more policy‐related features and activities than Jewish schools. Arab schools also have more features and activities that support the developing personal skills Area of Action: health education and staff training and empowerment. Arab schools were more likely to involve the community in health‐promotion activities than Jewish schools. However, Arab and Jewish schools did not differ in the number of environment‐related activities

**TABLE 3:** Health promotion areas of action in Arab and Jewish schools and by religious sectors among Jewish schools.

Strategy	Arab sector (*N* = 304)	Jewish sector (*N* = 331)				Comparison Total Arab vs. Jews	Comparison Secular Arab vs. Jews	Comparison School type
	Total (secular)	Total (*N* = 331)	Secular (*n* = 169)	Religious (*n* = 72)	Ultraorthodox (*n* = 90)			
	M (SD)	M (SD)	M (SD)	M (SD)	M (SD)	*t*/*z*/*χ* ^2^ (*d*)	*t*/*z*/*χ* ^2^ (*d*)	*F*/*H*/*χ* ^2^ (*η* ^2^)
Policy	7.42 (1.93)	5.86 (2.08)	6.76 (1.85)	5.56 (1.80)	4.50_c_ (1.87)	9.81*** (0.78)	3.42*** (0.33)	85.98*** (0.22)
Environment	7.42 (1.96)	7.20 (2.19)	8.02 (1.97)	7.00 (2.12)	5.83_c_ (1.93)	1.34 (0.11)	−3.21*** (−0.31)	31.69*** (0.09)
Developing personal skills								
Education^†^	2.05 (0.73)	1.64 (0.74)	1.89_a_ (0.72)	1.11 (0.74)	0.70_c_ (0.76)	6.70*** (0.56)	1.92 (0.2)	99.46*** (0.15)
Teaching staff training and empowerment^a^	1.56 (0.62)	1.13 (0.78)	1.37 (0.71)	1.68 (0.67)	1.13_c_ (0.54)	7.14*** (0.61)	2.99** (0.3)	86.57*** (0.16)
Involve community^b^	92.5%	57.6%	76.5%	61.3%	28.0%	72.75***	17.99***	47.07***

^a^ Non‐parametric comparison (Mann–Whitney test/Kruskal–Wallis test). Means with different subscripts within a row significantly differ at the *p* < 0.05 level by Tukey's HSD post hoc test (ANOVA) or Bonferroni correction (Kruskal–Wallis test).

^b^ Chi‐square analysis.

** *p* < 0.01.

*** *p* < 0.001.

Regarding different religious sectors within the Jewish schools, the study results indicated that secular schools have significantly more policy and environment‐related Areas of Action than religious schools, which in turn have significantly more policy and environment‐related features and activities than ultraorthodox schools. Similarly, significant differences exist in the number of activities that support staff training and empowerment. Secular schools have significantly more activities that support health education and staff training and empowerment than religious schools, which in turn have significantly more activities that support health education and staff training and empowerment than ultraorthodox schools. Moreover, secular schools were the most likely to involve the community in health‐promotion activities (parents (79%), health professionals (82%), and NGOs or volunteer organizations (65%)), followed by religious schools (parents (9%), health professionals (9%), and NGOs or volunteer organizations (26%)) and ultraorthodox schools (parents (10%), health professionals (5%), and NGOs or volunteer organizations (9%)).

### 3.3 Correlations Between Health Promoting Strategies and Socioeconomic Rank

Spearman correlations were computed to examine whether the number of health‐promoting features and activities in the school is associated with the socioeconomic rank of the local authority in which the school resides. In the total sample of schools, higher socioeconomic rank was associated with more environment‐related features and activities (*r* = 0.13, *p* < 0.01), but less with staff training and empowerment (*r* = −0.15, *p* < 0.001) and community involvement (*r* = −0.16, *p* < 0.001).

The Arab‐minority local authorities tend to have a lower socioeconomic rank compared to localities from the Jewish majority in the current sample and in general. Jewish schools (M = 4.91, SD = 1.70) resided in municipalities with a significantly higher socioeconomic rank than Arab schools (M = 3.26, SD = 1.21; *z* = 13.08, *p* < 0.001). Therefore, we also computed the correlations between socioeconomic rank and health‐promoting features and activities separately for Jewish and Arab schools. In Arab schools, a higher socioeconomic rank was associated with less staff training and empowerment (*r* = −12, *p* < 0.05) and a lower likelihood of community involvement (*r* = −0.19, *p* < 0.01). Regarding the other Areas of Action, no significant relationship was found between socioeconomic status of Arab schools and activities in the areas of policy, environment, and health education.

In contrast, in Jewish schools, higher socioeconomic rank was associated with more features and activities related to policy (*r* = 0.19, *p* < 0.001), environment (*r* = 0.29, *p* < 0.001), and health education (*r* = 0.26, *p* < 0.001), as well as a greater likelihood of community involvement (*r* = 0.14, *p* < 0.05). This suggests an interaction between ethnicity and health promotion activities in schools.

Multiple logistic regression analysis revealed several key factors associated with HPS status and specific strategy implementation (Table 4). Arab schools, secular Jewish schools, and elementary schools demonstrated a significantly higher likelihood of being a recognized HPS, with these three factors collectively explaining 27.5% of the variance.

**TABLE 4:** Multiple logistic regression analysis of factors associated with HPS status and areas of action implementation in school.

								Developing personal skills logistic regression	
	Health promoting schools logistic regression	Policy linear regression			Environmental linear regression			Teaching staff training and empowerment	Education
	OR (95% CI)	*B*	*β*	*t*	*B*	*β*	*t*	OR (95% CI)	OR (95% CI)
Socioeconomic Rank	0.97 (0.86–1.11)	0.03	0.02	0.56	0.13	0.107	2.38*	−0.03 (−0.08, 0.02)	0.02 (−0.02, 0.06)
Ethnicity	2.85 (1.78–4.56)	0.73	0.17	3.39***	−0.29	−0.070	−1.32	0.07 (−0.12, 0.26)	0.10 (−0.06, 0.27)
Religiosity	1								
Secular	2.97 (1.82–4.84)***	—						1	
Religious		−2.21	−0.36	−8.06***	−2.22	−0.37	−7.91***	−0.69 (−0.99, −0.40)*	−0.52 (−0.76, −0.27)***
Ultraorthodox	1.83 (0.99–2.29)	−1.15	−0.17	−4.26***	−0.85	−0.13	−3.09**	−0.22 (−0.47, 0.04)	−0.15 (−0.36, 0.06)
School stage	1								
Elementary	0.38 (0.17–0.88)***	—						1	
Middle school	2.77 (1.84–4.16)***	−0.19	−0.04	−1.14	−0.77	−0.167	−4.35***	−0.03 (−0.19, 0.12)	0.18 (0.05, 0.31)*
Constant	1.16 (0.57–2.36)	5.89							
*R* ^2^/Nagelkerke *R* ^2^	0.27	0.23***			0.15***				
Adjusted *R* ^2^		0.22***			0.14***				

*Note*: *** *p* < 0.001. ** *p* < 0.01. * *p* < 0.05.

Regarding specific health promotion strategies: Arab and secular schools were more likely to apply policy strategies. Schools from higher socioeconomic communities, secular schools, and elementary schools showed greater implementation of environmental strategies. Secular schools demonstrated better implementation of staff training and empowerment activities compared to religious schools. Middle schools were more likely to implement educational strategies than elementary schools.

These findings highlight clear demographic and institutional patterns in health promotion adoption across the school system, with ethnicity, religiosity, school level, and socioeconomic status emerging as significant predictors of both overall HPS status and specific strategy implementation.

## 4 Discussion

Although the Israeli Ministries of Health and Education required all schools to be designated as Health Promoting Schools (HPS) by 2020, the findings indicate that only about half achieved formal recognition. Notably, nearly half of the non‐HPS schools reported engaging in health promotion activities aligned with the Ottawa Charter's principles. This suggests that the core values of health promotion have become embedded in the broader educational culture, extending beyond the boundaries of formal certification. Similar diffusion of health promotion practices beyond official frameworks has been documented in previous studies [16, 23, 24], emphasizing that formal recognition alone may not fully capture the scope of school‐based health activities [25, 26]. Consequently, scholars have called for flexible recognition models that acknowledge diverse modes of implementation [27, 28, 29].

Consistent with international evidence, this study demonstrated that comprehensive implementation of the Ottawa Charter's five Action Areas was associated with stronger engagement in health promotion. Programs in Australia and Taiwan have similarly shown that systematic integration of all Charter domains enhances health outcomes and quality of life [21, 30]. Broad application of these principles has been linked to behavioral and lifestyle improvements, particularly among higher socioeconomic groups [31]. This study assessed all Ottawa Charter Action Areas, based on the premise that full implementation is more effective than partial implementation. These results align with growing advocacy for holistic, context‐sensitive application of the Charter to maximize both health impact and community participation [21, 32, 33, 34, 35].

However, challenges such as limited resources and insufficient professional development persist [36], potentially constraining program sustainability. Although the present study did not assess student outcomes directly, prior research elsewhere has shown that schools implementing Ottawa Charter strategies achieve measurable improvements in student health behaviors [37, 38, 39]. Continued research should evaluate whether these patterns hold within the Israeli context.

Arab‐sector schools demonstrated higher engagement in the HPS initiative, with low‐SES Arab schools showing particularly strong adherence to Ottawa Charter strategies. This finding corresponds with literature describing elevated health needs in disadvantaged or developing communities [40]. Nonetheless, persistent disparities suggest that existing governmental frameworks insufficiently address the sociocultural realities of these populations. In contrast, ultraorthodox schools exhibited lower rates of HPS recognition and strategy implementation, consistent with prior studies identifying barriers such as limited health literacy, economic hardship, and cultural stigma [41]. These findings underscore the importance of incorporating cultural and structural competence, fostering partnerships with religious communities, and tailoring interventions to promote equity and inclusion.

Elementary schools showed higher rates of HPS adoption and Charter implementation than middle schools, emphasizing the value of early health promotion interventions. Prior research among elementary schools, highlights the role of principal leadership, structured training, and clear communication in sustaining these efforts [42]. Early initiation of primary prevention has been shown to enhance students' motivation, internalization of health knowledge, and ability to critically evaluate risky behaviors [43].

Within the Arab sector, an inverse association was found between SES and HPS recognition, whereas no such pattern emerged among Jewish schools, likely due to their generally higher SES. International research confirms that higher SES facilitates the adoption of HPS and Ottawa Charter strategies [32, 36]. Yet, despite substantial engagement of low‐SES Arab schools in HPS programs, Arab students continue to report poorer nutrition and greater environmental exposure than Jewish peers [44], mirroring global inequities observed in disadvantaged populations. Key contributing factors include lower per‐student funding, reduced parental health literacy, and limited access to supportive physical environments. When socioeconomic status is accounted for, achievement gaps between Jewish and Arab students narrow substantially, suggesting that disparities stem primarily from structural inequalities rather than cultural factors [45].

### 4.1 Implications for School Health Policy, Practice, and Equity

These findings collectively highlight that expanding and strengthening the Health Promoting Schools framework can serve as a practical mechanism to reduce health disparities between Jewish secular, religious and Arab students in Israel. Ensuring equitable allocation of resources, culturally adapted implementation, and sustained governmental commitment is essential for achieving long‐term health equity through the education system.

### 4.2 Strengths and Limitations

A major strength of this study is its comprehensive inclusion of nearly all schools in the Northern District, made possible by the region's centralized school health services. The Northern district of Israel includes around 43% of Jews and 53% of Arabs living in Israel. However, it is important to note that some schools may have reported a higher level of health promotion activity due to awareness of Ministry of Health expectations for HPS recognition, potentially introducing social desirability bias into their self‐reports. It should be noted that the nurses interviewing the principals are not employees of the education system but of the health system. This role configuration may have introduced information bias. Future research should use independent data collection, broaden geographic scope, and include diverse perspectives to better understand school health promotion. Objective measures of health promotion activities could help understand both the extent and the specific characteristics of health promotion implementation in schools. Moreover, since the research was conducted exclusively in schools within the Northern District, the generalizability of the findings to other regions in the country or internationally is limited. Future studies including diverse geographic and demographic contexts could enhance the applicability and relevance of the results across broader populations. Another limitation is that the study did not examine the effectiveness of using the Ottawa Charter Areas of Action as reflected in indicators such as students' behaviors and their health outcomes; however, other studies have provided this type of data [21, 30, 31]. Therefore, further research is needed to assess student‐level outcomes and develop innovative solutions to reduce disparities in health promotion across different populations.

## 5 Conclusions

Strengthening the implementation of the Ottawa Charter principles alongside formal recognition of HPSs is fundamental to advancing effective school health promotion. To achieve meaningful and sustainable progress, it is crucial to develop and implement policies that explicitly focus on equity, ensuring that all schools, particularly those in underserved and marginalized communities, have equal access and opportunity to promote health. Such equity‐oriented strategies must address structural barriers and be adaptable to the unique cultural and contextual needs of diverse populations. Without this focused approach, disparities in health promotion will likely persist, limiting the overall impact of school‐based health initiatives and health equity goals. Early intervention, especially in elementary schools, is crucial for establishing lifelong healthy behaviors and should be prioritized. A holistic approach, integrating supportive environments, community engagement, and aligned policies, is essential for successful implementation. Finally, the results from the Israeli study on health promotion implementation in schools hold important implications for multicultural and multiethnic educational contexts internationally. Similar to Israel's diverse Jewish and Arab school sectors, many countries operate schools with students from varied ethnic, cultural, and socioeconomic backgrounds. Hence, the implementation strategies and equity focus observed in Israel could inform the design and evaluation of health promotion programs in other multicultural educational systems worldwide, supporting improved health and educational outcomes across diverse student populations.

## Funding

The authors have nothing to report.

## Ethics Statement

The study was approved by the ethics committee of the Max Stern Yezrael Valley College (EMEK 2022–80).

## Conflicts of Interest

The authors declare no conflicts of interest.

## Data Availability

The data that support the findings of this study are available on request from the corresponding author. The data are not publicly available due to privacy or ethical restrictions.

## Appendix A

**TABLE A1:** Description of the research questionnaire variables.

Variable	Questions
Engagement in health promotion	Does the school engage in health promotion activities? Has your school been officially recognized as a health‐promoting school by the Ministry of Education in recent 3 years?
Activities according to the Ottawa Charter	
Policy	Is there a school charter that includes health issues? Is there a school policy regarding sports classes beyond the requirements set by the Ministry of Education? Are students allowed to use the sports hall outside school hours? Is there a leader/coordinator/focal point for health promotion in the school? Is there a steering committee on health promotion in the school? After receiving a vaccination or completing another task at school, does the student receive a candy or snack? Do children watch a smart screen, phone, television, or other devices while eating meals at school? Which of the following foods and beverages are available within the school premises?
Environment	Does the school have sports facilities? Does the school have drinking facilities? Do teachers or visitors smoke in the school? Does the school have a vegetable garden on its premises?
Developing personal skills: – Health education – Teaching staff training and empowerment	Is health content integrated into the education school curriculum? In what ways does your school engage in health‐related activities (health days, health week)? Does the school curriculum include lessons on smoking prevention? Are interactive activities held at the school (any activity that is not a regular lesson) on topics related to health, such as nutrition, physical activity, screen use, sleep, and smoking prevention? Have the teachers participated in motivational and educational health‐related training in the last 3 years?
Community involvement	Does the school involve the community (parents, community nurses, etc.) in health‐related activities?
Background data	Ethnicity of the students (Arabs or Jews), education sector (religiosity), school stage and SES of the town or village?
